# 4-Hydroxycoumarin inhibits biofilm formation and enhances antibiotic efficacy against *Escherichia coli* O157:H7

**DOI:** 10.3389/fmicb.2026.1934000

**Published:** 2026-10-01

**Authors:** Hongxing Zhang, Jing Xu, Yaxin Zhou, Zhijin Zhang, Zixuan Shang, Bing Li, Yubin Bai, Jiyu Zhang

**Affiliations:** 1 Key Laboratory of New Animal Drug Project of Gansu Province, Key Laboratory of Veterinary Pharmaceutical Development of the Ministry of Agriculture and Rural Affairs, New Veterinary Drugs Engineering Research Center of Gansu Province, Key Laboratory of New Veterinary Drugs Engineering of CAAS, Laboratory Animal Science Data Center of Gansu Province, Key Laboratory of Animal Genetics and Breeding on Tibetan Plateau, Ministry of Agriculture and Rural Affairs, Lanzhou Institute of Husbandry and Pharmaceutical Sciences of CAAS, Lanzhou, Gansu, China; 2 College of Life Science and Food Engineering, Hebei University of Engineering, Handan, Hebei, China

**Keywords:** 4-hydroxycoumarin, biofilm, *Escherichia coli* O157:H7, LuxS, QS

## Abstract

**Background:**

Conventional bactericidal antibiotics often fail to eradicate Escherichia coli O157:H7 (*E. coli* O157) infections due to biofilm-mediated antimicrobial tolerance, and monotherapy can inadvertently drive resistance selection. This study aimed to identify compounds capable of modulating LuxS/AI-2-related phenotypes and to evaluate their potential to disrupt biofilms and enhance antibiotic efficacy against *E. coli* O157.

**Methods:**

The effects of 4-HC on biofilm formation, extracellular polymeric substance (EPS) biosynthesis, and flagella-mediated motility were assessed in vitro. Antibiotic potentiation was evaluated through combination assays across multiple antibiotic classes. In vivo efficacy was tested in a murine systemic infection model, with qualitative histological observation used to evaluate splenic, hepatic, and ileal tissue damage.

**Results:**

4-HC disrupted biofilm formation by suppressing EPS biosynthesis and flagella-mediated motility, thereby destabilizing biofilm architecture and impairing bacterial dissemination. 4-HC acted as a broad-range potentiator for most antibiotics tested, with the most pronounced effect observed in combination with fosfomycin sodium. This combination reduced systemic bacterial burden and restored intestinal barrier function *in vivo*. In the murine model, the 4-HC–fosfomycin regimen improved survival and markedly attenuated splenic necrosis and ileal tissue damage, with efficacy superior to monotherapy.

**Conclusion:**

4-HC disrupts biofilm formation and enhances antibiotic efficacy against *E. coli* O157 infection, supporting the strategy of combining biofilm-modulating adjuvants with conventional antibiotics to combat antimicrobial resistance. However, rigorous safety evaluation is required before clinical translation.

## 1 Introduction

In recent years, the escalating burden of antibiotic resistance has been attributed not only to horizontal gene transfer and point mutations but also to the sophisticated biofilm architectures that bacteria assemble within specific microenvironments (Ciofu et al., 2022). Biofilm formation represents a bacterial adaptive strategy that drives chronic infection and treatment failure through enhanced adhesion, elevated pathogenicity, and antibiotic tolerance exceeding planktonic cells by orders of magnitude (Costerton et al., 1999). According to the National Institutes of Health (NIH), more than 60% of bacterial infections and up to 80% of chronic infections are biofilm-associated (Jamal et al., 2018), thereby underscoring the urgent need for adjunctive strategies that disrupt biofilm architecture and potentiate the efficacy of existing bactericidal agents (Hawas et al., 2022). Enterohemorrhagic *Escherichia coli* O157:H7 (*E. coli* O157) is a globally dominant foodborne pathogen whose ability to form dense biofilms within the host gastrointestinal tract and on food-processing surfaces underlies its persistence, transmissibility, and resistance to antimicrobial therapy (Nemati et al., 2025). Its public health impact is amplified by notable environmental resilience, enabling persistence throughout the agrifood continuum (Bridges et al., 2018). Conventional antibiotic therapies for *E. coli* O157 infections exert selective pressure that accelerates the emergence of antimicrobial resistance (Schroeder et al., 2002), and the propensity of *E. coli* O157 to form biofilms serves as a critical mechanism underlying antimicrobial failure (Wang et al., 2020). Biofilm-mediated persistence shields the pathogen from host immune surveillance while simultaneously serving as a reservoir for antimicrobial-resistant strains, thereby amplifying the public health burden (Jeon et al., 2025; Yang et al., 2025). Beyond the clinical contexts, this same persistence facilitates recurrent contamination throughout the food production chain and elevates the risk of severe infection upon exposure (Wang et al., 2014; Schwartz et al., 2011). Consequently, overcoming these therapeutic constraints requires moving beyond conventional monotherapy toward adjuvant-based approaches. For instance, combining natural bioactive compounds with antibiotics represents a promising strategy to achieve synergistic eradication while mitigating resistance selection (Zhang et al., 2025; Xu et al., 2026). This biofilm tolerance arises from a confluence of mechanisms, including diffusion barriers conferred by the extracellular polymeric substances and quorum sensing (QS)-mediated coordination of biofilm development and metabolic adaptation (Høiby et al., 2010; Karygianni et al., 2020; Wang et al., 2022). Within this network, the LuxS/autoinducer-2 (AI-2) pathway serves as a central regulatory circuit in *E. coli* O157, governing motility (Kalia et al., 2019), matrix production (Hufnagel et al., 2015), and biofilm development (Solano et al., 2014). *LuxS*-dependent AI-2 signaling mediates the transition from motile planktonic cells to sessile, matrix-embedded biofilms, while also modulating biofilm maturation and persistence (Wang et al., 2019). Disrupting this axis thus represents a strategy that both undermines biofilm integrity and resensitizes bacteria to antimicrobials, making it an attractive target for adjuvant-mediated sensitization. However, small molecules capable of selectively interfering with this signaling axis remain largely unexplored.

Building on this premise, 4-hydroxycoumarin (4-HC) was identified through screening as a potent candidate capable of disrupting biofilm formation and compromising biofilm-mediated colonization of *E. coli* O157. 4-HC shares the benzopyrone scaffold of warfarin, an anticoagulant with an established clinical profile (Rost et al., 2004; Kasperkiewicz et al., 2020). Beyond its canonical role in haemostasis, the coumarin scaffold has attracted attention for diverse bioactivities, including anti-inflammatory and anticancer effects (Balewski et al., 2021). This established background makes 4-HC a plausible candidate for drug repurposing as a biofilm-disrupting adjuvant. We hypothesized that 4-HC interferes with the LuxS/AI-2 pathway, thereby modulating the regulatory cascade governing extracellular polysaccharide (EPS) production and biofilm maturation, and resensitizing *E. coli* O157 to conventional antibiotics. To test this, a *luxS*-eGFP reporter platform was integrated with comprehensive phenotypic assays to evaluate QS inhibition, biofilm architecture, and metabolic activity. The potential of 4-HC to potentiate antibiotics was subsequently assessed, and this combination was validated in a murine *E. coli* O157 infection model using fosfomycin sodium and 4-HC. While Lee et al. (2014) previously demonstrated that coumarin derivatives reduce *E. coli* O157:H7 biofilm formation and virulence, the present study extends this line of investigation in three key respects. First, we implemented a *luxS*-eGFP reporter-based high-throughput workflow (Z’ = 0.955) to profile LuxS/AI-2-associated phenotypes and prioritize 4-HC as a candidate modulator. Second, we identified 4-HC–fosfomycin sodium as a novel pairing among eight screened antibiotics. Third, we provide the first *in vivo* validation in a murine systemic infection model demonstrating enhanced antibiotic efficacy. These advances support the continued development of coumarin-based antimicrobial adjuvants by linking phenotypic screening to an optimized antibiotic pairing with *in vivo* validation.

## 2 Materials and methods

### 2.1 Materials and bacteria

The 4-hydroxycoumarin used in this study was purchased from MedChemExpress. Polymyxin B (≥98%), tetracycline hydrochloride (≥98%), fosfomycin sodium (≥98%), gentamicin sulfate (≥98%), azithromycin (≥98%), cefquinome (≥98%), meropenem (≥98%), and streptomycin (≥98%) to be tested and used in this study were purchased from Shanghai Yuanye Biotechnology Co., Ltd. (Shanghai, China). All antibiotics were of analytical grade or higher and used as received. 4-HC was dissolved in dimethyl sulfoxide (DMSO) to yield a 40 mg/mL stock solution. The final DMSO concentration in the LB medium was maintained below 1%. For control experiments, an equal volume of DMSO without the compound was added to account for the solvent effect.

*Escherichia coli* O157 (ATCC 43895; stx1 + stx2+) was obtained from the American Type Culture Collection (ATCC). Luria-Bertani (LB) broth and Luria-Bertani agar (LA), purchased from HuanKai Microbial (Guangdong, China), were used for the cultivation of all *E. coli* strains. *Vibrio Harveyi BB170* (*V. harveyi BB170*) and *V. Harvey BB152* (*V. harveyi BB152*) are gifts from researcher Xiang’an Han (Shanghai Veterinary Research Institute, Chinese Academy of Agricultural Sciences). The growth medium consisted of AB medium (0.05 M MgSO₄, 0.3 M NaCl, and 0.2% vitamin-free Casamino Acids per liter) supplemented with 1 mM L-arginine, 1% phosphate buffer (pH 7.2), and 1% glycerol.

### 2.2 *LuxS-eGFP* reporter strain construction

The *luxS* fragment was amplified by PCR using *luxS-F*/*luxS-R* (Table 1) with genomic DNA of *E. coli* O157 as the template following standard molecular cloning procedures (Sambrook and Russell, 2016; Xu et al., 2026). The pUC-19 plasmid served as the vector backbone. The *luxS* and *eGFP* fragments were then fused by overlap extension PCR and ligated into pUC-19 following restriction digestion with T4 DNA ligase, yielding the recombinant plasmid pUC-*luxS*-*eGFP*. The construct was then transformed into *E. coli* DH5α competent cells. After colony screening and plasmid extraction, the recombinant plasmid was confirmed by sequencing and subsequently electrotransformed into *E. coli* O157 competent cells to generate the *luxS*-*eGFP* reporter strain.

**Table 1:** Primer sequences for PCR analysis.

Target genes		Sequences (5′-3′)
*luxS*	Forward	GGATCCTCTAGAATGCCGTTGTTAGATAGCTTCACAG
	Reverse	TGCTCACCCTCCGATGTGCAGTTCCTGCAACTTCTC
*luxS*-*eGFP*	Forward	TACGCCAAGCTTATGCCGTTGTTAGATAGCTTCACAG
	Reverse	GGATCCTCTAGATTACTTGTACAGCTCGTCCATGCC

### 2.3 LuxS/AI-2 pathway screening

The *luxS-eGFP* reporter strain was cultured at 37 °C with shaking (200 rpm) until the optical density at OD600 reached 0.6. Protein expression was induced by the addition of isopropyl-*β*-D-thiogalactopyranoside (IPTG) to a final concentration of 1 mM, followed by a further incubation period of 4 h. Subsequently, the cultures were treated with the QS inhibitor furanone C30 at 60 μM (He et al., 2012; Suzuki et al., 2024). To validate the screening platform, a negative control group was established, yielding a Z’ factor of 0.955 and confirming the robustness of the *luxS-eGFP* reporter system. For small-molecule screening, test compounds were added to the bacterial culture simultaneously with IPTG and incubated for 4 h. Subsequently, 200 μL of each culture was transferred to a black opaque 96-well plate, with three technical replicates per group. Fluorescence intensity was measured using a multimode microplate reader (excitation: 488 nm; emission: 509 nm), and the assay was repeated three times independently. Compounds exhibiting significant inhibition of *luxS-eGFP* fluorescence were selected for subsequent experiments.

Single colonies of *E. coli* were inoculated into LB broth and activated through two successive passages. The cultures were adjusted to OD600 = 1.0 and subcultured 1:100 into fresh LB broth in the presence of the *luxS* inhibitor and test compounds, followed by co-culture for 16 h. After centrifugation at 12,000 × *g* for 5 min, the supernatant was filtered through a 0.22 μm pore-size membrane to obtain the cell-free supernatant (CFS) (Park et al., 2017).

*V. harveyi BB170* was cultured in AB medium and activated through two successive passages. The culture was then diluted 1:100 into fresh AB medium and incubated at 30 °C with shaking (160 rpm) until the OD_600_ reached approximately 1.0 (Zhang et al., 2025). Subsequently, the cells were diluted 1:2500 in AB medium. For the AI-2 bioassay, 20 μL of CFS from *E. coli* cultures was mixed with 180 μL of the diluted *V. Harvey Yi BB170* suspension in a white opaque 96-well plate. After incubation in the dark at 30 °C for 4 h, bioluminescence intensity was measured using a multimode microplate reader. CFS from an overnight culture of *V. Harvey Yi BB152* served as the positive control, and all assays were performed in triplicate.

### 2.4 Biofilm inhibition

Biofilm inhibition and eradication assays with 4-HC were assessed using the crystal violet (CV) staining assay (O'toole, 2011). *E. coli* O157 was initially cultured at 37 °C for 24 h. Following this, the bacterial suspension was standardized to an OD₆₀₀ of 0.01 and dispensed into white 96-well plates (Corning Costar® 3,599, Corning, Kennebunk, Maine, USA) containing 4-HC at concentrations ranging from 6.25 μg/mL to 400 μg/mL. After a subsequent 24-h incubation at 37 °C, non-adherent cells were removed by three washes with PBS (pH 7.2). The remaining biofilms were fixed with methanol, stained with 0.1% (w/v) CV for 30 min, and destained with tap water. Bound dye was solubilized in 150 μL of 95% ethanol, and absorbance was measured at 595 nm. All assays were performed in triplicate.

### 2.5 Biofilms eradication

*Escherichia coli* O157 was initially cultured statically at 37 °C for 24 h. The resulting bacterial suspension was then adjusted to an OD₆₀₀ of 0.01, and 150 μL aliquots were transferred to 96-well microplates (Corning Costar® 3,599, Corning Inc., USA). Following a 24-h incubation at 37 °C to permit biofilm formation, the wells were gently washed three times with PBS (pH 7.2). Pre-formed biofilms were subsequently exposed to 4-HC at concentrations ranging from 25 μg/mL to 400 μg/mL and incubated for an additional 24 h. After treatment, the medium was aspirated, and the biofilms were washed three times with sterile PBS. The adherent cells were fixed with methanol for 30 min and stained with 0.1% (m/v) crystal violet (CV) for 10 min. Unbound dye was removed by three PBS washes, and the remaining CV was solubilized in 150 μL of 95% ethanol. Absorbance was measured at 595 nm using a microplate reader. All assays were performed in triplicate.

### 2.6 Bacterial growth and viability assays

The effect of 4-HC on the growth of *E. coli* O157 was monitored using a microplate reader. Briefly, overnight cultures were diluted 1:100 into fresh LB medium containing 4-HC at final concentrations ranging from 100 to 400 μg/mL. The cultures were then dispensed into 96-well flat-bottom plates and incubated at 37 °C with continuous shaking. The optical density (OD₆₀₀) was measured automatically every 2 h over a 12 h period. Wells containing uninoculated medium served as blanks for background correction, while bacterial cultures without 4-HC served as controls. To further assess bacterial viability, CFU assays were performed in parallel. At designated time points (0, 2, 4, 6, 8, 10, and 12 h), aliquots were collected from each treatment group, serially diluted in sterile PBS, and plated onto LB agar. Colonies were counted after incubation at 37 °C for 24 h. All experiments were performed in triplicate.

### 2.7 Scanning electron microscopy (SEM) analysis

Biofilm morphology was examined via scanning electron microscopy (SEM) with slight modifications to established protocols (Bai et al., 2025). Concisely, bacterial suspensions were inoculated into 96-well plates containing cell culture slides and exposed to 100–400 μg/mL 4-HC. Following a 24-h incubation at 37 °C to facilitate biofilm formation, the samples were gently washed three times with sterile PBS to remove planktonic bacteria and loosely adherent cells. The cell culture slides were then carefully extracted from the wells and fixed with 2.5% glutaraldehyde in PBS at 4 °C for 2 h. After three PBS washes, samples were dehydrated through a graded ethanol series (30, 50, 70, 90, and 100%, 15 min per step), followed by critical point drying. The dried samples were sputter-coated with gold for 60 s and examined using an FEI Versa 3D scanning electron microscope (Thermo Fisher Scientific, Waltham, MA, USA) at an accelerating voltage of 5.0 kV. All the assays were conducted in three independent replicates (*n* = 3).

### 2.8 Bacterial motility

The impact of 4-HC on *E. coli* motility was assessed using a semi-solid agar assay (Lee et al., 2014). Briefly, an overnight culture was standardized to an OD₆₀₀ of 1.0. Subsequently, 1 μL of the bacterial suspension was spotted onto 0.3% semi-solid LB agar plates supplemented with varying concentrations of 4-HC (100–400 μg/mL). Following incubation at 37 °C for 24 h, motility was quantified by measuring the migration halo diameter relative to the control group. Each experiment was performed in triplicate.

### 2.9 EPS production

The quantification of extracellular polysaccharides (EPS) was performed using a ruthenium red staining assay according to the previous method (Adnan et al., 2020). Briefly, *E. coli* O157 cultures exposed to varying concentrations of 4-HC were seeded into 96-well plates and incubated at 37 °C for 24 h to allow biofilm formation. Following three washes with PBS, 200 μL of 0.01% (w/v) ruthenium red solution was added to each well (with blank wells receiving staining solution only). After a 60 min dark incubation at 37 °C, the unbound dye was transferred to a fresh plate, and the absorbance was read at 450 nm. Three independent biological replicates were conducted for each treatment. The EPS inhibition rate was subsequently calculated based on the following formula:


EPSInhibition(%)=((AP−AS)/(AP−AB))×100%

AB: Absorbance of the blank control solution (staining solution only, no bacteria);

AS: Absorbance of the sample solution (4-HC treated *E. coli* O157);

AP: Absorbance of the positive control (untreated *E. coli*) solution.

### 2.10 Combined effects of 4-HC and antibiotics on bacterial viability

The effect of 4-HC on the activity of conventional antibiotics was evaluated following the previous method (Xu et al., 2026). Briefly, *E. coli* O157 was cultured overnight and subsequently diluted to an OD600 of 0.01. These bacterial suspensions were then exposed to sub-inhibitory concentrations of antibiotics (1/2, 1/4, and 1/8 MIC) either in the absence or presence of 4-HC at 400 μg/mL. Following incubation at 37 °C for 16–18 h, the metabolic activity of the mixed cultures was quantified using the Alamar Blue assay. Each experiment was performed in triplicate.

### 2.11 *In vivo* efficacy of 4-HC and fosfomycin sodium

Female 6-week-old C57BL/6 J mice (18–22 g) were procured from the Lanzhou Veterinary Research Institute, Chinese Academy of Agricultural Sciences. All animal procedures were conducted in strict accordance with the guidelines for the care and use of laboratory animals and were approved by the Institutional Animal Care and Use Committee of the Lanzhou Institute of Husbandry and Pharmaceutical Sciences of CAAS (Approval No. 2025–16). Following a 3-day acclimatization period, mice were randomized into four groups (*n* = 12 per group): Control (vehicle), Fosfomycin Sodium, 4-HC, and a combination group (4-HC + Fosfomycin Sodium). *E. coli* O157 was cultured overnight and subcultured once before use. The bacterial suspension was adjusted to a 0.5 McFarland standard (equivalent to 1.0 × 10^8^ CFU/mL) and further concentrated to a final density of 1.5 × 10^9^ CFU/mL. On day 1, mice were intraperitoneally challenged with 0.2 mL/10 g of the bacterial suspension. One hour post-infection, treatments were administered orally by gavage at 0.2 mL/10 g body weight. Fosfomycin sodium was prepared at a concentration of 2.5 mg/mL in the dosing vehicle, yielding 0.5 mg/10 g body weight per administration. The solution was administered alone or in combination with 4-HC (40 mg/kg) to the appropriate groups. Survival was recorded daily over a 7-day period, and Kaplan–Meier survival curves were generated. On day 7, the remaining mice were euthanized via cervical dislocation under isoflurane anesthesia (RWD, China). Target tissues were harvested and preserved in appropriate fixatives or frozen for subsequent analysis, including bacterial load quantification (plate counting method). For the Model group, bacterial burden data are presented as *n* = 3, comprising 2 day-7 survivors and 1 animal that died on day 6, from which tissues were collected aseptically at the time of death. For all other groups, bacterial loads were quantified from 3 randomly selected day-7 survivors. Animals that died before day 6 were recorded for survival analysis and subjected to gross necropsy for macroscopic pathological documentation. Sterile tissue harvest for CFU assay was not performed on these earlier non-survivors.

### 2.12 Histopathological and immunofluorescence analysis

Spleen, liver, and ileum tissues were fixed in 4% paraformaldehyde (Servicebio, China), dehydrated in a graded ethanol series, and cleared in xylene. The tissues were subsequently embedded in molten paraffin, sectioned into 5–8 μm slices using a rotary microtome, and stained with hematoxylin and eosin (H&E) for histopathological evaluation (Guo et al., 2023). For immunofluorescence analysis of tight junction proteins, sections were deparaffinized, subjected to antigen retrieval, and blocked with serum for 30 min to reduce non-specific binding. The sections were then incubated overnight at 4 °C with primary antibodies against ZO-1 and occludin, followed by incubation with the corresponding secondary antibody in the dark at room temperature for 1 h. Nuclei were counterstained with DAPI, and the slides were mounted for imaging.

### 2.13 Statistical analysis

All statistical evaluations and graphical representations were generated using GraphPad Prism (version 10.0, GraphPad Software, San Diego, CA, USA). Data normality and homogeneity of variances were assessed prior to analysis. Data are presented as mean ± standard error of the mean (SEM) of *n* independent biological replicates. For growth curve analyses, two-way RM ANOVA with Greenhouse–Geisser correction and Dunnett’s *post hoc* test was used. For other experiments involving multiple groups at a single time point, statistical significance was assessed using one-way ANOVA followed by Tukey’s HSD *post hoc* test. For comparisons between two groups, Student’s t-test was used. For survival analysis, the Log-rank (Mantel-Cox) test was used. Exact *p* values are provided in the figures, tables, and text. Statistical significance was defined as *p* < 0.05.

## 3 Results

### 3.1 Suppression of LuxS-mediated signaling by 4-HC

To discover potential QS inhibitors interfering with the LuxS/AI-2 system in *E. coli* O157, a *luxS-eGFP* reporter system was successfully constructed by inserting the *luxS-eGFP* fusion fragment derived from *E. coli* O157 into the pUC-19 vector, which was subsequently introduced into the host strain via electroporation. The expression of the fusion gene was induced by IPTG to verify the reliability of the screening model. To validate the responsiveness of this system, the known QS inhibitor furanone C30 was employed as a positive control. As shown in Figure 1A, furanone C30 treatment significantly suppressed the fluorescence intensity of the reporter strain, which was detected by a multi-mode microplate reader. Subsequently, high-throughput screening of the compound library of our laboratory was conducted using this validated reporter platform. 4-HC was identified as a potent inhibitor capable of significantly decreasing *luxS-eGFP* fluorescence (Figure 1B). To further elucidate the functional impact of this compound on QS, the influence of 4-HC on AI-2 activity was assessed using the biosensor strain *V. harveyi BB170*. The results indicate that 4-HC treatment led to a significant decrease in AI-2-mediated luminescence (Figure 1C). To exclude the possibility that the observed effects were due to bacterial toxicity, the MIC and growth curve of 4-HC against *E. coli* O157:H7 were determined. The MIC of 4-HC was > 400 μg/mL, and no significant differences in OD600 or CFU counts were observed at tested concentrations (Supplementary Figures S1, S2). These results indicate that 4-HC reduces luxS-eGFP fluorescence and AI-2-mediated luminescence independently of gross toxicity, suggesting potential interference with QS-related processes.

**Figure 1:**
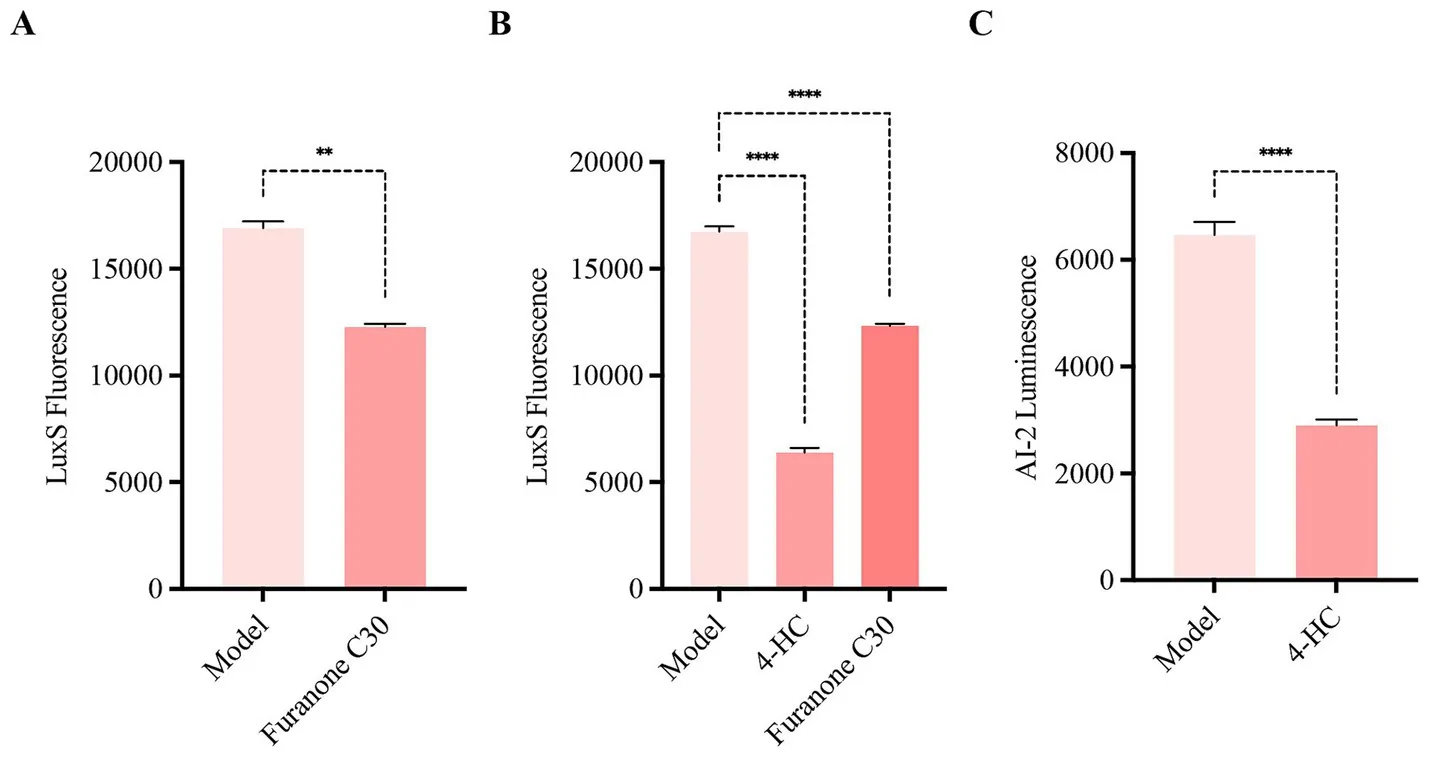
Functional characterization of 4-hydroxycoumarin (4-HC) using a *luxS-eGFP* reporter. **(A)** Fluorescence of the *luxS-eGFP* reporter strain following furanone C30 treatment (60 μM). **(B)** Inhibition of *luxS-eGFP* fluorescence by 4-HC (400 μg/mL). **(C)** The level of AI-2 following 4-HC treatment (400 μg/mL). Values are absolute, non-normalized readings (*n* = 3 per group; Data represent means ± SEM; *p < 0.05, **p < 0.01, ***p < 0.001, ****p < 0.0001).

### 3.2 Inhibition of biofilm formation and motility in *E. coli* O157 by 4-HC

As shown in Figure 2A, the quantitative analysis of biofilm inhibition rate revealed a clear dose-dependent response to 4-HC treatment. As the concentration of 4-HC increased from 6.25 μg/mL to 400 μg/mL, the biofilm inhibition rate gradually rose, reaching a maximum inhibition of approximately 70% at the highest concentration tested. Notably, even at relatively low concentrations (25–50 μg/mL), 4-HC exhibited substantial inhibitory activity, reducing biofilm biomass by nearly 30%. Figure 2B further demonstrates the efficacy of 4-HC against pre-formed biofilms. Treatment with increasing concentrations of 4-HC led to a progressive increase in biofilm eradication, with the highest concentration (400 μg/mL) achieving an eradication rate of over 50%, indicating that 4-HC can effectively disrupt established biofilms. Figure 2C provides visual evidence from SEM to complement the quantitative data. In the control group, *E. coli* O157 formed dense, confluent biofilms with numerous bacterial cells tightly packed together. In contrast, treatment with 100 and 200 μg/mL 4-HC resulted in a visibly sparser biofilm architecture, characterized by increased intercellular spacing and reduced cell aggregation. At higher concentrations (400 μg/mL), the biofilms were severely disrupted, with only scattered individual cells or small clusters remaining adherent to the surface. These morphological changes suggest that 4-HC interferes with bacterial adhesion and microcolony formation. Beyond disrupting biofilm development, 4-HC also significantly inhibited bacterial motility. Figure 2D shows representative images of swarming motility assays, where the diameter of the swarming zone is directly proportional to bacterial motile capacity. In the absence of 4-HC (control), *E. coli* O157 exhibited extensive swarm expansion, forming large, well-defined rings. However, as the concentration of 4-HC increased, the swarming zones became progressively smaller. Figure 2E quantifies this observation, demonstrating a statistically significant, dose-dependent reduction in swarm diameter. Specifically, the swarm diameter decreased from approximately 11 mm in the control group to less than 7 mm at 400 μg/mL 4-HC (*p* < 0.0001), confirming that 4-HC effectively suppresses the swarming behavior of *E. coli* O157. Collectively, these findings demonstrate that 4-HC exerts potent inhibitory effects on multiple biofilm-related phenotypes in *E. coli* O157, including biofilm formation, mature biofilm disruption, and swarming motility.

**Figure 2:**
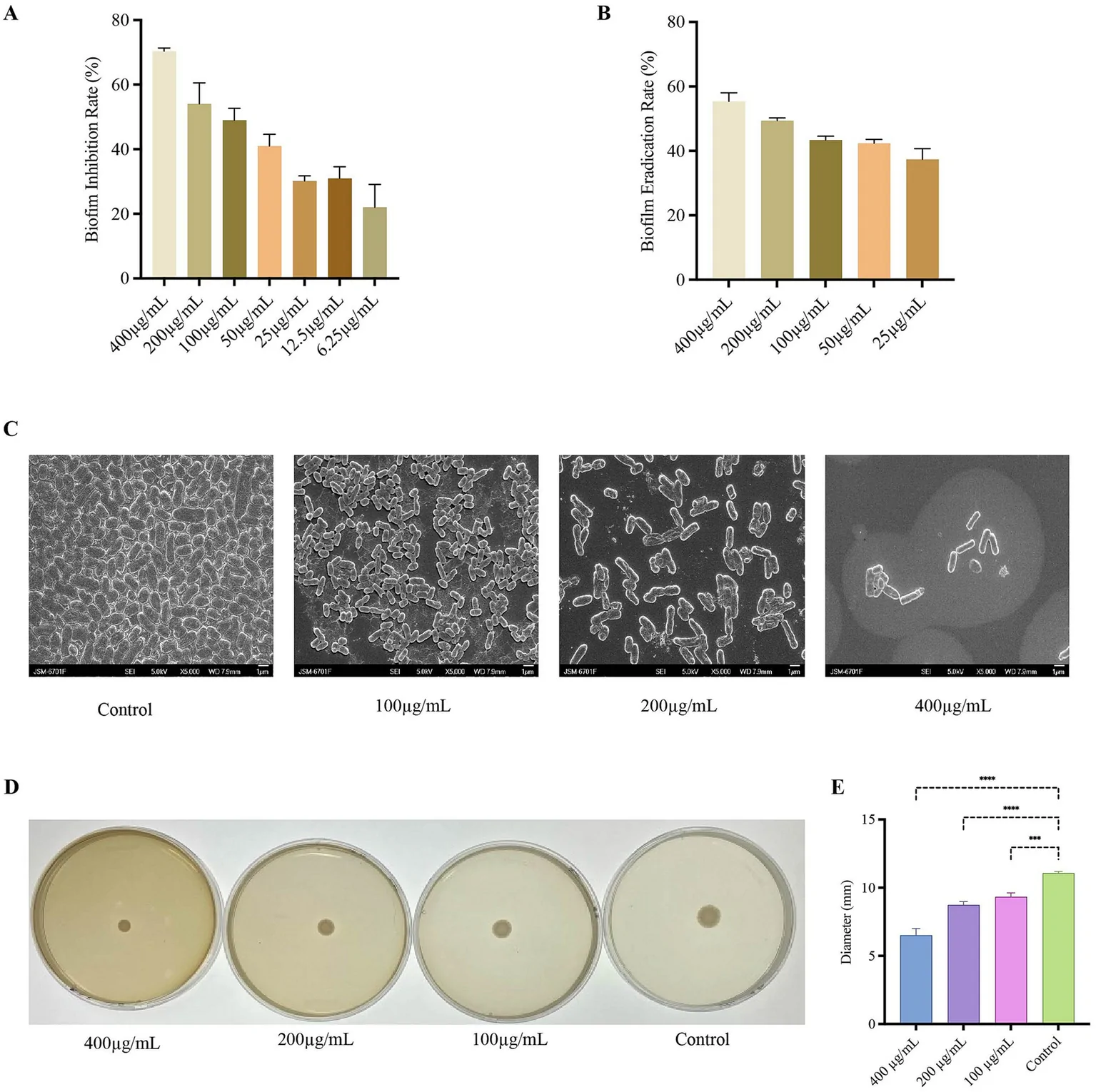
4-Hydroxycoumarin (4-HC) inhibits biofilm formation and motility in *E. coli O157*. **(A)** Biofilm inhibition rate of *E. coli* O157 after treatment with increasing concentrations of 4-HC. **(B)** Biofilm eradication rate of pre-formed biofilms of *E. coli* O157 following 4-HC treatment. **(C)** Scanning electron micrographs of *E. coli* O157 treated with different concentrations of 4-HC (Scale bar = 1 μm). **(D)** Representative images of swarming motility assays, showing the inhibitory effect of 4-HC on bacterial swarm expansion. **(E)** Quantification of swarm diameters, indicating a dose-dependent inhibition of *E. coli* O157 swarming motility by 4-HC (*n* = 3 per group; Data represent means ± SEM; *p < 0.05, **p < 0.01, ***p < 0.001, ****p < 0.0001).

### 3.3 Inhibition of EPS production in *E. coli* O157 by 4-HC

Figure 3 illustrates the inhibitory effect of 4-HC on EPS production in *E. coli* O157. As depicted in the bar graph, treatment with 4-HC resulted in a significant, dose-dependent reduction in EPS levels. Administration of 400 μg/mL 4-HC led to the most pronounced inhibitory effect, reducing EPS production by approximately 90%. Even at a halved concentration (200 μg/mL), 4-HC maintained strong efficacy, inhibiting EPS production by nearly 70%. These quantitative findings demonstrate that 4-HC effectively disrupts EPS production in a dose-responsive manner.

**Figure 3:**
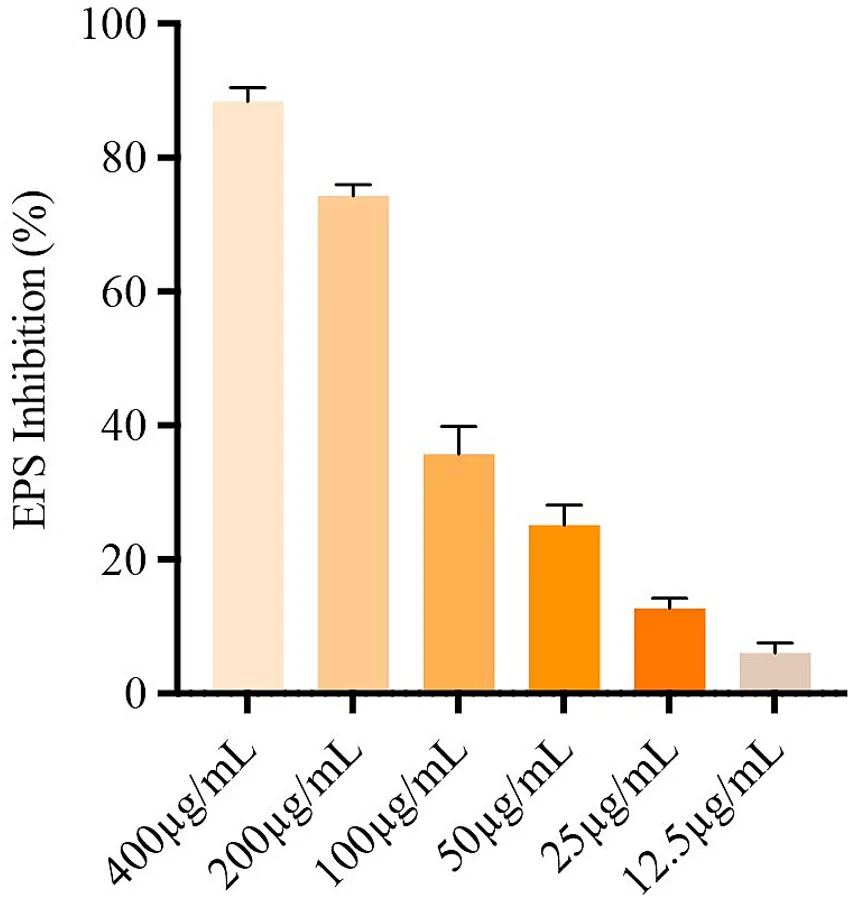
4-Hydroxycoumarin (4-HC) inhibited extracellular polysaccharides (EPS) production in *E. coli* O157 in a dose-dependent manner (*n* = 3 per group; Data represent means ± SEM).

### 3.4 Combination effects of 4-HC with antibiotics

Figure 4 presents the effects of eight common antibiotics, either alone or in combination with a fixed concentration of 4-HC, on the viability of *E. coli* O157 at sub-inhibitory concentrations. For fosfomycin sodium (Figure 4A), the combination with 4-HC resulted in a significant reduction in bacterial viability across all tested concentrations. At 1/2 MIC, the viability dropped from approximately 55% (monotherapy) to below 5% (combination). Similarly, at 1/4 and 1/8 MIC, the viability was significantly lower in the presence of 4-HC compared to the antibiotic alone, with reductions of roughly 40%. Figure 4B (tetracycline hydrochloride) and Figure 4C (cefquinome) display comparable trends. In both cases, the addition of 4-HC to the antibiotic treatments led to a marked decrease in viable cell counts. For tetracycline, the most dramatic drop occurred at 1/2 MIC, where viability fell from around 50% to below 25%. Cefquinome showed a similar pattern, with the combination treatment reducing viability to approximately 16% at 1/2 MIC, compared to over 35% for the monotherapy. Streptomycin (Figure 4D) also exhibited enhanced efficacy in the presence of 4-HC. At 1/2 MIC, viability decreased from about 45% to around 20%, and this trend persisted at lower concentrations, although the absolute viability values were higher. At 1/4 and 1/8 MIC, the viability was also significantly lower in the presence of 4-HC compared to the antibiotic alone, with reductions of roughly 40%. Meropenem (Figure 4E), gentamicin (Figure 4F), and azithromycin (Figure 4G) followed the same pattern. The combination treatments resulted in significantly lower viability percentages than the antibiotics alone at all concentrations. For meropenem, the reduction was particularly stark at 1/2 MIC, with viability plummeting from roughly 55 to 15%. For gentamicin and azithromycin, while the antibiotics alone exhibited a typical dose-dependent decline in efficacy (viability rising from 1/2 to 1/8 MIC), the co-administration of 4-HC consistently maintained substantial bactericidal activity even at these sub-inhibitory doses. In stark contrast, polymyxin B (Figure 4H) exhibited a marked antagonistic interaction. In the combination group, bacterial viability increased dramatically from roughly 20% (antibiotic alone) to approximately 70% at 1/2 MIC, indicating that 4-HC significantly counteracted the bactericidal effect of polymyxin B.

**Figure 4:**
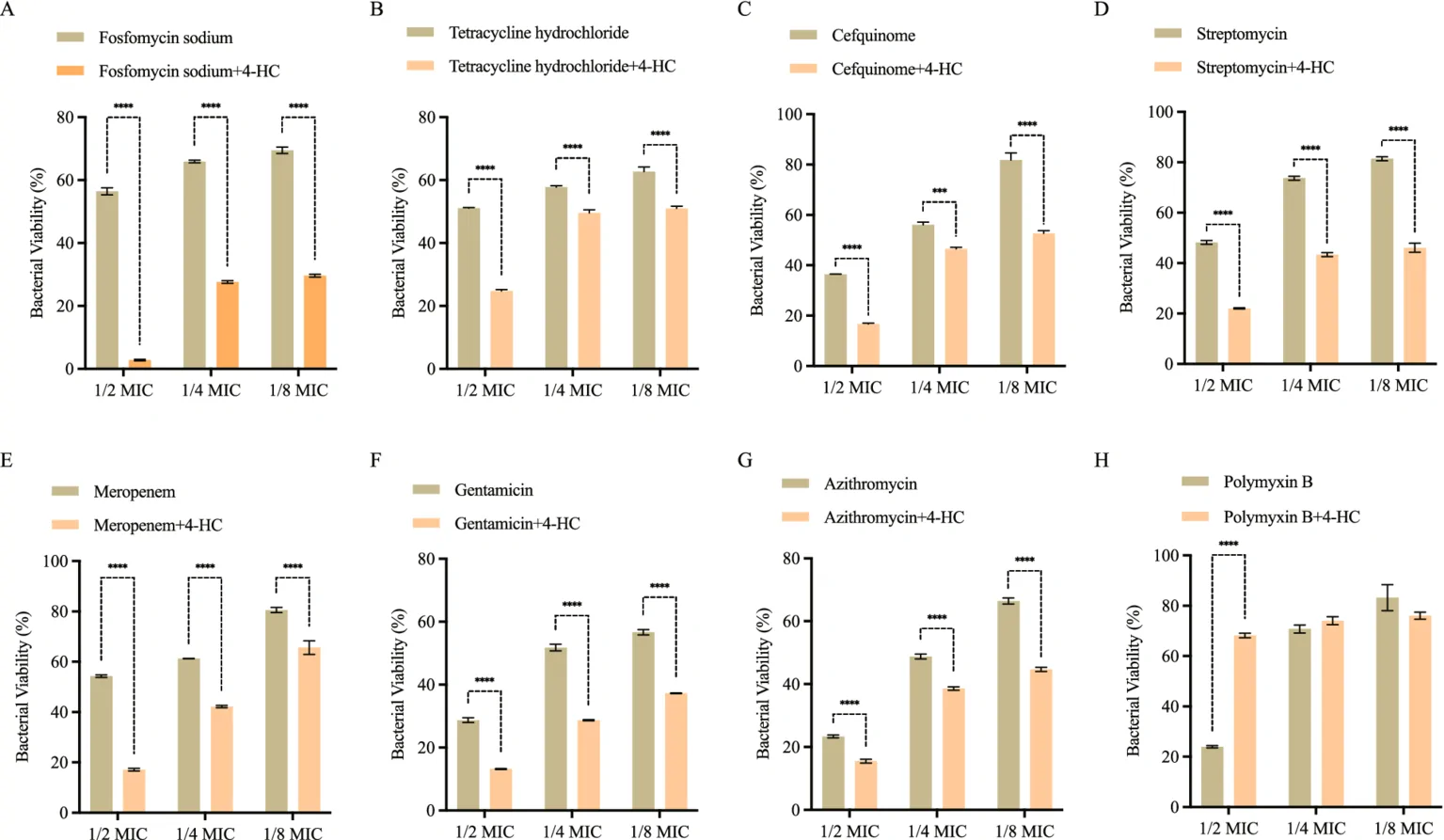
Effects of antibiotic-4-Hydroxycoumarin (4-HC) combination on bacterial viability. Bar graphs **(A–H)** show the viability of *E. coli* O157 treated with eight common antibiotics (fosfomycin sodium, tetracycline hydrochloride, cefquinome, streptomycin, meropenem, gentamicin, azithromycin, and polymyxin B) at sub-inhibitory concentrations (1/2, 1/4, and 1/8 MIC), either alone or in combination with a fixed concentration of 4-HC (*n* = 3 per group; Data represent means ± SEM; *p < 0.05, **p < 0.01, ***p < 0.001, ****p < 0.0001).

### 3.5 Efficacy of 4-HC in combination with fosfomycin sodium

Based on the *in vitro* interaction profiles summarized in Figure 4, 4-HC demonstrated a marked capacity to potentiate the efficacy of fosfomycin sodium, driving bacterial viability down to nearly 5% at the 1/2 MIC level. This significant enhancement of the reduction in bacterial viability prompted the selection of the 4-HC-fosfomycin sodium regimen for further evaluation in an *in vivo* infection model. Figure 5A presents the Kaplan–Meier survival curves for the different treatment groups. While administration of fosfomycin sodium alone resulted in a modest survival benefit compared to the untreated model group, the combination therapy with 4-HC and fosfomycin sodium showed improved survival. Notably, the survival rate in the combination group reached 75% by day 7, representing a further improvement in protection compared to monotherapy. However, pairwise log-rank comparison between the combination group (75% survival, 3/12 deaths) and fosfomycin monotherapy (50% survival, 6/12 deaths) did not reach statistical significance (*p* = 0.2457), likely reflecting limited group sizes (*n* = 12 per group). In line with the improved survival observed, quantitative analysis of bacterial loads in the target organs (Figures 5B,C) revealed the superior efficacy of the combination treatment. In both the spleen and liver, the combination therapy induced a substantial reduction in *E. coli* O157 colonization compared to all other groups. Specifically, fosfomycin sodium monotherapy reduced splenic bacterial loads from approximately 9.5 log₁₀ CFU (model) to around 5.0 log₁₀ CFU (*p* < 0.0001). However, the addition of 4-HC further potentiated this effect, driving the bacterial load down to approximately 4.5 log₁₀ CFU. Similarly, in the liver, the combination treatment achieved a nadir of approximately 4.0 log₁₀ CFU/g, which was significantly lower than both the model group (~9.0 log₁₀ CFU/g) (*p* < 0.0001) and the fosfomycin sodium group, demonstrating a potentiating effect on bacterial burden reduction.

**Figure 5:**
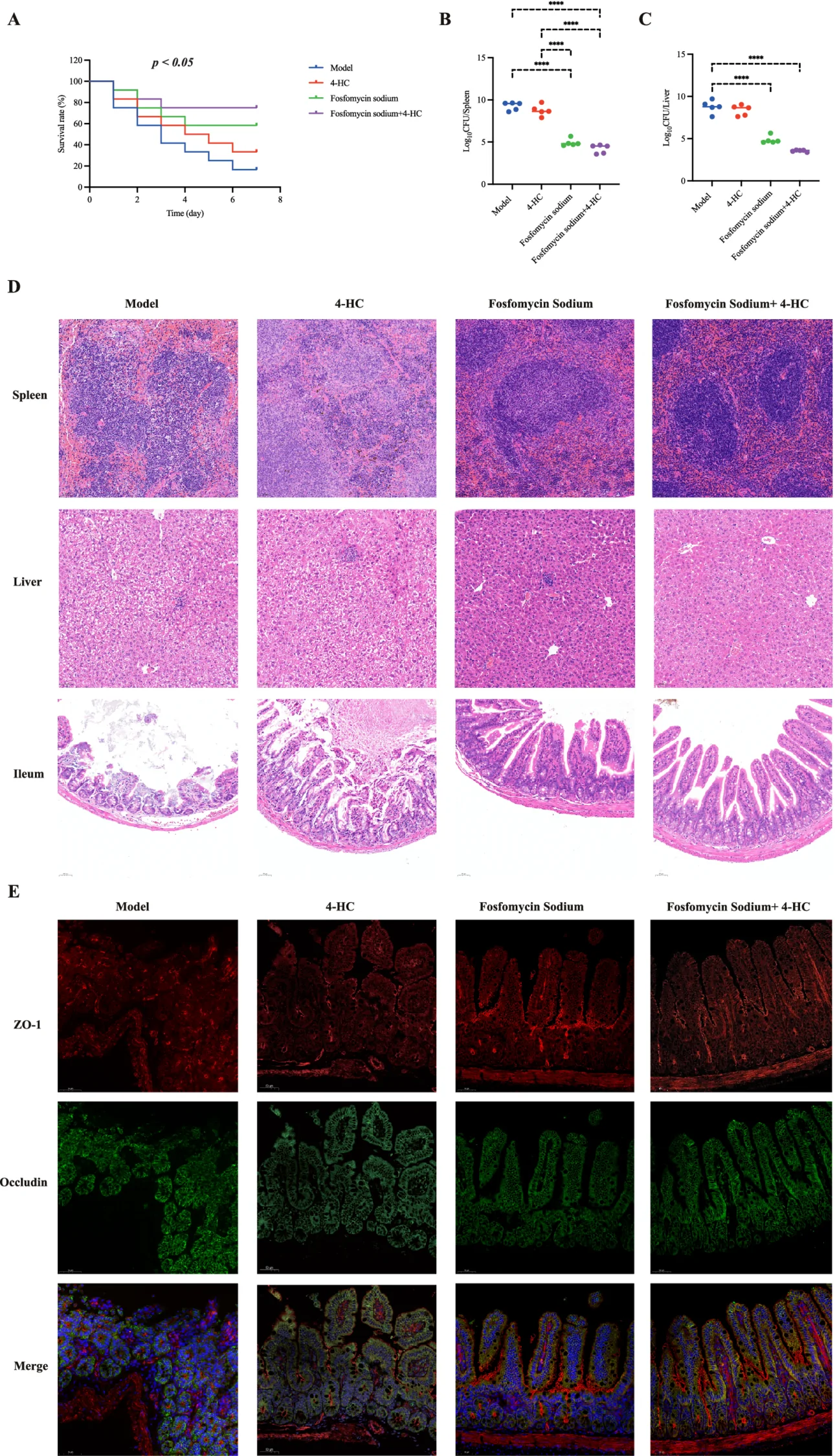
The efficacy of 4-hydroxycoumarin (4-HC) in combination with fosfomycin sodium against *E. coli* O157 infection in a murine model. **(A)** Survival rate of mice following *E. coli* O157 infection under different treatments. Mice (*n* = 12 per group) were treated with vehicle (Model), 4-HC alone, fosfomycin sodium alone, or their combination. **(B)** Bacterial loads in the spleen of *E. coli* O157-infected mice after different treatments. Mice were treated with vehicle (Model), 4-HC alone, fosfomycin sodium alone, or their combination. **(C)** Bacterial loads in the liver of *E. coli* O157-infected mice after different treatments (*n* = 3 per group; Data represent means ± SEM; *p < 0.05, **p < 0.01, ***p < 0.001, ****p < 0.0001). **(D)** Hematoxylin and eosin (H&E) staining of spleen, liver, and ileum from *E. coli* O157-infected mice after treatment. **(E)** Immunofluorescence staining of tight junction proteins (ZO-1 and Occludin) in ileum tissues with different treatments.

Furthermore, H&E staining (Figure 5D) revealed severe pathological damage in the model group across the spleen, liver, and ileum, characterized by extensive inflammatory cell infiltration, architectural disorganization, and focal necrosis. In the spleen, extensive and poorly demarcated hemorrhagic necrotic foci were observed alongside severe disruption of the normal splenic corpuscle architecture, indicating substantial infection-induced injury. In the liver, the model group exhibited disordered hepatic cord architecture with extensive inflammatory cell infiltration and punctate necrosis around central veins and portal areas, confirming severe infection-induced hepatitis. In the ileum, significant damage to the small intestinal mucosal structure was observed, manifested specifically as villous atrophy, epithelial detachment, and disruption of the tissue structure. Treatment with 4-HC alone moderately reduced inflammatory cell infiltration and hepatocyte damage, showing a better outcome than the model, but it did not significantly alter the bacterial burden or substantially restore tissue architecture. In contrast, fosfomycin sodium monotherapy markedly diminished hemorrhagic necrosis and considerably improved splenic structure, while also effectively suppressing hepatic inflammation and promoting partial recovery of intestinal villi. Most notably, the combination of 4-HC and fosfomycin sodium produced the most pronounced therapeutic effect across all tissues examined. In the spleen, hemorrhagic necrotic lesions were nearly absent, splenic corpuscles appeared clearly defined with only mild residual inflammation, and the overall morphology closely resembled that of normal spleen tissue. In the liver, the combination group exhibited substantially reduced inflammatory infiltration, promoted recovery of hepatic cord organization, and markedly ameliorated tissue damage, representing the most favorable outcome among all treatments. In the ileum, the combination therapy further significantly alleviated the intestinal mucosal tissue damage and promoted the recovery of villus morphology to a near-normal state, outperforming the protective effects observed with fosfomycin sodium monotherapy alone. Finally, immunofluorescence staining of ileum tissues (Figure 5E) supported the protective effect of the combination on the intestinal barrier. While *E. coli* O157 infection severely disrupted the expression and continuity of the tight junction proteins ZO-1 and occludin in the model group, treatment with fosfomycin sodium alone partially restored their expression. Most strikingly, the combination of 4-HC and fosfomycin sodium robustly enhanced the abundance and structural integrity of both ZO-1 and Occludin, exhibiting superior efficacy compared to both the model group and all monotherapy groups.

## 4 Discussion

### 4.1 Identification of 4-HC as a potential modulator of QS-related phenotypes

The LuxS/AI-2 QS system represents a strategically attractive target for biofilm-disrupting approach against enterohemorrhagic *E. coli* O157 (Xu et al., 2025). Disrupting *LuxS* activity offers a dual advantage by compromising biofilm matrix production and motility, thereby distinguishing it from conventional bactericidal agents that drive resistance selection. A critical methodological consideration in QS inhibitor screening is the choice of reporter platform. In this study, a *luxS-eGFP* transcriptional fusion system was engineered by cloning the promoter and coding sequence of *luxS* from *E. coli* O157 into pUC-19 under IPTG-inducible control, followed by electroporation into the parent strain. This design provides two strategic advantages over constitutive reporters. First, IPTG induction standardizes baseline fluorescence, thereby minimizing clonal variation (Binder et al., 2016). Second, the eGFP fusion enables real-time reporting of *luxS* transcriptional activity, which more accurately reflects functional QS output than conventional mRNA-based proxies (Schauder et al., 2001; Bai et al., 2026). The robustness of this platform was validated using furanone C30, a well-established QS inhibitor originally identified (He et al., 2012). The substantial fluorescence suppression elicited by this positive control confirmed the reliability of our reporter strain for detecting QS inhibitor activity. Consequently, high-throughput screening of our laboratorial compound library identified 4-HC as a potent candidate. This compound mediated a pronounced attenuation of *luxS-*eGFP fluorescence, establishing its potential as a compound with QS-related activity. To ascertain whether this transcriptional suppression translated into functional impairment of AI-2 secretion and perception, the well-established *V. harveyi* BB170 biosensor assay was employed (Bonnie et al., 2006). This pronounced suppression of AI-2 bioluminescence suggests that 4-HC interferes with both *luxS* transcription and intercellular signaling pathways. However, biosensor toxicity or quenching by 4-HC cannot be excluded as the cause of reduced luminescence. In summary, we established a sensitive *luxS*-eGFP *E. coli* O157 reporter system and used it to identify 4-HC as a potent QS-interfering compound. Nevertheless, we acknowledge that the pUC19-based, IPTG-inducible system does not fully recapitulate the native regulatory environment of luxS. The high-copy nature of the plasmid may influence gene dosage and cellular responses to stress. Therefore, while our findings provide a strong proof-of-concept for 4-HC as biofilm-disrupting adjuvant with potential QS-related effects, future validation using chromosomally integrated reporters will be necessary to confirm these observations under physiologically relevant conditions. Furthermore, while these findings confirm that 4-HC suppresses *luxS* reporter activity and AI-2 signaling, it remains to be determined whether this observed suppression is attributable to direct inhibition of the LuxS protein or indirect modulation via alterations in bacterial metabolism. Further mechanistic investigations, such as LuxS enzymatic activity assays, molecular docking, and protein-ligand interaction studies, are required to clarify the precise mode of action of 4-HC. Regardless, the phenotypic suppression of QS signaling by 4-HC provides a strong foundation for its potential as a biofilm-disrupting adjuvant against *E. coli* O157:H7.

### 4.2 Biofilm and motility impairment by 4-HC

The data presented herein demonstrate that 4-HC exerts potent biofilm-disrupting effects against *E. coli* O157, disrupting both the establishment and maintenance of biofilm communities. The approximately 70% inhibition of *de novo* biofilm formation at 400 μg/mL demonstrates that 4-HC disrupts early surface colonization, specifically by impairing initial adhesion and microcolony development. These structural alterations were directly visualized by SEM, which captured the transition from compact, confluent control biofilms to disaggregated cellular networks upon 4-HC treatment. This disruption of structural integrity is consistent with the expected phenotypic consequences of LuxS/AI-2 interference. In *E. coli* O157, AI-2 signaling is indispensable for the coordinated expression of curli amyloid fibers and cellulose synthases, the primary extracellular polymeric substances that constitute the biofilm matrix (Flemming and Wingender, 2010). By potentially suppressing *luxS* signaling, 4-HC may block the signals required for EPS production, preventing the shift from initial attachment to stable biofilm adhesion. Parallel to recent LuxS/AI-2-targeted screens that successfully identified anti-biofilm candidates (Bai et al., 2025; Bai et al., 2026; Xu et al., 2026), a particularly notable attribute of 4-HC is its capacity to disrupt established mature biofilms. This compound introduces a novel coumarin scaffold that couples QS-related interference with structural biofilm disruption, expanding the chemical diversity available for biofilm-targeted adjuvant development. Conventional antibiotics predominantly target core biosynthetic processes, such as cell wall synthesis and protein translation, which are typically downregulated (Ballén et al., 2022). In stark contrast, 4-HC targets the luxS/AI-2 axis, a regulatory hub that retains sustained metabolic activity to orchestrate matrix turnover and dispersal signaling, thereby circumventing a common mechanism of biofilm tolerance. The suppression of swarming motility further elucidates the biofilm-disrupting breadth of 4-HC. By impairing flagellum-dependent motility (Sauer et al., 2022), 4-HC limits the ability of *E. coli* O157 to reach and colonize surfaces, thereby reducing the pool of planktonic cells available to initiate new biofilm communities and enhancing the accessibility of antibiotics to sessile populations. Given that LuxS-derived AI-2 functions as a pivotal signal promoting flagellar gene expression, the intracellular reduction of AI-2 by 4-HC would be expected to suppress the flagellar transcriptional cascade. This clinical relevance stems from the necessity of robust motility for both mucosal invasion and gut colonization, as well as its role in driving the hypermotile phenotype characteristic of *E. coli* O157 during intestinal colonization (Sun et al., 2022). Taken together, these results define the biofilm-disrupting capacity of 4-HC, wherein disruption of the LuxS/AI-2 axis concurrently disrupts biofilm cohesion and impairs bacterial motility. The dose-dependent suppression of EPS provides a key mechanistic basis for the biofilm dispersal observed in Figure 2. The ~90% reduction in EPS production at 400 μg/mL is a substantial phenotypic shift, indicating that 4-HC does not merely prevent cell aggregation but significantly impairs the biosynthetic capacity of the bacteria to produce the materials required for community infrastructure (Limoli et al., 2015). The robust dose-dependent suppression, evidenced by ~70% inhibition at 200 μg/mL, suggests a high degree of target engagement and potent *in vitro* activity. Notably, even at the lowest concentration tested (12.5 μg/mL), a modest but detectable suppressive effect on EPS production was observed (~5%). Taken together, these findings position 4-HC as a selective disrupter of biofilm-associated biosynthesis. By limiting EPS production, 4-HC deprives the biofilm of essential structural scaffolds, thereby compromising its defensive integrity.

### 4.3 Combination effects of 4-HC with antibiotics

The combinatorial screening revealed that 4-HC functions as a broad-spectrum potentiator of conventional antibiotics against *E. coli* O157, despite failing to potentiate polymyxin B combinations. Across eight antibiotics evaluated, encompassing inhibitors of cell wall synthesis (fosfomycin, cefquinome, and meropenem), protein translation (tetracycline, and azithromycin), and aminoglycosides (streptomycin and gentamicin), supplementation with 4-HC at subinhibitory concentrations consistently potentiated antibiotic-mediated reductions in bacterial viability. This potentiating effect was most pronounced with fosfomycin and meropenem, where 4-HC potentiated the reduction in bacterial viability by over 40% at 1/2 MIC, effectively shifting the antibiotic response from a persistence to a bactericidal range. The mechanistic basis for this broad-spectrum potentiation may be rooted in the multifaceted disruption of bacterial physiology mediated by 4-HC. First, by disrupting the biofilm matrix and reducing EPS production, 4-HC eliminates the physical diffusion barrier and the protected niche that shelters persister cells, thereby enhancing antibiotic penetration and bioavailability. Second, the suppression of motility and the metabolic shifts associated with potential QS modulation likely prevent the formation of slow-growing, antibiotic-tolerant subpopulations. Third, for cell wall targeting antibiotics such as fosfomycin and meropenem, 4-HC could create a state of heightened vulnerability. In contrast, the marked antagonism observed with polymyxin B invites mechanistic interrogation beyond the phenotype. Multiple mechanisms may contribute to this antagonism. One plausible explanation is that 4-HC–mediated LuxS/AI-2 disruption may alter outer membrane composition (e.g., lipid A acylation, surface charge alterations, OmpF/OmpC expression), shifting net surface charge toward reduced negativity and reducing polymyxin B electrostatic interaction with lipid A (Olaitan et al., 2014; Fernandez-Ciruelos et al., 2023; Davin-Regli et al., 2024). Alternatively, QS-dependent outer membrane restructuring may interfere with the ability of polymyxin B to displace bridging Mg^2+^/Ca^2+^ at lipopolysaccharide inter-bridging sites, through enhanced divalent cation binding density that stabilizes the outer membrane despite polymyxin exposure (Yu et al., 2015; Mohapatra et al., 2021). These hypotheses may collectively reflect coordinated outer membrane remodeling driven by QS disruption. While the combination of 4-HC with conventional antibiotics represents a promising experimental approach, we acknowledge that such adjunctive combinations are not universally beneficial. The antagonism observed with polymyxin B underscores the necessity of rational adjuvant-antibiotic pairing. Unlike dual-bactericidal combinations that may exert compounded selective pressure, 4-HC functions as a non-bactericidal potentiator at subinhibitory concentrations, thereby reducing the risk of resistance development. Nevertheless, the translation of these findings into clinical settings requires further validation in diverse clinical isolates and complementary infection models.

### 4.4 Combined *in vivo* efficacy of 4-HC and fosfomycin sodium in murine models

The successful application of *in vitro* potentiation into the murine systemic infection model supports the *in vivo* relevance of 4-HC as an antibiotic adjuvant for *E. coli* O157. Among the eight antibiotics screened, fosfomycin sodium showed the strongest potentiation by 4-HC, emerging as the lead candidate for *in vivo* evaluation. The numerical improvement in survival supports the notion that modulation of LuxS/AI-2 signaling and EPS suppression observed *in vitro* may contribute to enhanced bacterial burden reduction *in vivo*. Whereas the combination group showed numerically improved survival relative to monotherapy, larger cohort studies are warranted to formally confirm this benefit. Organ profiling revealed that combining 4-HC with fosfomycin reduced the bacterial burden in both the spleen and liver relative to monotherapy. Specifically, bacterial burden quantification was performed on 3 randomly selected day-7 survivors per group, except the Model group, which included 2 day-7 survivors and 1 interval-death sample from an animal that died on day 6 (tissues collected aseptically at death). Animals that died before day 6 were not included in CFU analysis. Those with the highest bacterial burdens likely died earliest and were most concentrated in the Model group, meaning they may have been excluded from the bacterial burden comparison. Accordingly, this sampling asymmetry likely overestimates the apparent magnitude of the treatment effect when comparing bacterial burdens across groups. This potentiating effect may reflect multiple pathways, 4-HC biofilm-disrupting activity and motility inhibition likely contribute to enhanced antibiotic accessibility and may promote bacterial burden reduction from the liver and spleen. Although the CFU data are subject to the sampling asymmetry described above, histological findings support the protective effects of the combination. Histopathological analysis revealed severe tissue damage in untreated controls, characterized by extensive inflammatory infiltrates, architectural disorganization, and focal necrosis across the spleen, liver, and ileum. While monotherapy with 4-HC offered modest protection, it failed to significantly reduce the systemic bacterial burden, yet attenuated tissue inflammation. Conversely, fosfomycin monotherapy mitigated organ damage by suppressing bacterial loads, yet residual inflammation and incomplete structural recovery persisted. The 4-HC and fosfomycin combination demonstrated complementary effects on bacterial burden reduction and tissue protection. The combination markedly attenuated splenic necrosis, substantially improved hepatic integrity, and largely restored ileal villi to a near physiological state, outperforming monotherapy. This capacity to preserve mucosal barrier function and alleviate pathology in a systemic infection model offers support for combining biofilm-disrupting strategies with standard antibiotics to combat systemic *E. coli* O157 infections and mitigate the associated inflammation. Notably, *E. coli* O157 infection disrupts tight junction proteins (ZO-1, occludin), leading to increased gut permeability and contributing to systemic complications (Shifflett et al., 2005; Kuo et al., 2021). While fosfomycin monotherapy partially restored ZO-1/Occludin expression, the 4-HC-fosfomycin combination produced a near complete recovery of tight junction architecture. Beyond its effects on bacterial phenotypes, 4-HC may confer *in vivo* benefit through direct anti-inflammatory activity. Previous studies have demonstrated that 4-HC exhibits anti-inflammatory properties (Luchini et al., 2008; da Fonsêca et al., 2025). Consistent with this, 4-HC monotherapy in the present study attenuated tissue inflammation without reducing bacterial burden, suggesting that the anti-inflammatory effects are at least partially independent of its antimicrobial activity. Thus, the observed efficacy of the 4-HC–fosfomycin combination likely reflects complementary mechanisms: fosfomycin provides direct bacterial killing, while 4-HC contributes through combined phenotypic modulation (e.g., EPS suppression, motility inhibition) and direct tissue anti-inflammatory effects. While this study demonstrates the *in vivo* efficacy of 4-HC as an antibiotic adjuvant, we acknowledge several limitations. First, comprehensive safety evaluations of 4-HC remain incomplete. Although the 40 mg/kg dose falls within established well-tolerated preclinical ranges (Luchini et al., 2008; da Fonsêca et al., 2025; Sadan et al., 2026), key toxicological assessments were not performed. Given that 4-HC shares the benzopyrone scaffold of warfarin, and related coumarin derivatives could be associated with hepatotoxicity (Adler et al., 1986; Arora and Goldhaber, 2006; Garg et al., 2020), comprehensive toxicological evaluations are warranted in subsequent preclinical studies. These should include hepatic assessment (e.g., serum ALT, AST, ALP, and total bilirubin), coagulation profiling (e.g., PT, APTT, and platelet function), and hemolysis assays, performed in both uninfected and combination-treated animals. Additionally, Shiga toxin production and stx transcription should also be evaluated in treatment animals. These host toxicity and bacterial virulence assessments represent a critical translational barrier that must be addressed before clinical application of 4-HC–antibiotic combinations can be considered. Second, while the systemic infection model provided quantitative assessment of bacterial clearance and systemic pathology, we acknowledge that *E. coli* O157:H7 is primarily an intestinal pathogen. An intestinal colonization model would more closely mimic the natural route of infection, and future studies should incorporate such models to validate the findings under conditions that more closely mimic human infection. Third, although histological observations revealed consistent group-level differences in tissue damage and recovery, a formalized blinded quantitative scoring system was not applied in the current study. Future investigations should incorporate blinded quantitative histopathological scoring to provide more rigorous statistical validation.

In summary, consistent with the expected consequences of LuxS/AI-2 interference, 4-HC concurrently suppresses the biosynthesis of extracellular polymeric substances, thereby inhibiting *de novo* biofilm formation and compromising the structural integrity of mature communities, while also impairing flagella-mediated motility. Future structural studies are required to elucidate the molecular basis of this activity, which will facilitate the rational design of more potent derivatives. Furthermore, validation in clinical *E. coli* isolates and complementary infection models is warranted to confirm these findings. Collectively, these data delineate a strategy wherein 4-HC acts as a potentiator with fosfomycin to mitigate systemic infection and tissue damage, offering a potential alternative approach to address antimicrobial resistance.

## 5 Conclusion

In this study, 4-HC was identified as a compound with potential to modulate LuxS/AI-2-related phenotypes in *E. coli* O157. By suppressing extracellular polymeric substance biosynthesis and flagella-mediated motility, 4-HC disrupted biofilm integrity and impaired bacterial dissemination. Crucially, 4-HC acts as a broad-range antibiotic potentiator, with its combination with fosfomycin sodium appearing to reduce systemic bacterial burden (subject to the CFU sampling limitations discussed) and improving intestinal barrier function in a murine systemic infection model. This mechanism may involve modulation of the LuxS/AI-2 QS axis, which concurrently inhibits biofilm-related phenotypes and promotes the restoration of intestinal barrier function. Nonetheless, the precise mode of action of 4-HC on the LuxS/AI-2 signaling cascade remains to be fully elucidated, and further studies are warranted to validate this combination in clinical *E. coli* O157 isolates. In summary, combining 4-HC with antibiotics represents a potential strategy against systemic *E. coli* O157 infections. By targeting QS-related phenotypes rather than essential biosynthetic pathways, 4-HC avoids direct bactericidal pressure. This may reduce resistance selection while restoring susceptibility to conventional drugs, supporting the development of biofilm-disrupting adjuvants for use alongside standard antimicrobial therapy. However, given the lack of comprehensive toxicological data, rigorous safety evaluation is required before clinical translation of 4-HC–antibiotic combinations.

## Data Availability

The raw data supporting the conclusions of this article will be made available by the authors, without undue reservation.

## References

[ref1] Adler E. Benjamin S. B. Zimmerman H. J. (1986). Cholestatic hepatic injury related to warfarin exposure. Arch. Intern. Med. 146, 1837–1839. 3753126

[ref2] Adnan M. Patel M. Deshpande S. Alreshidi M. Siddiqui A. J. Reddy M. N. . (2020). Effect of *Adiantum philippense* extract on biofilm formation, adhesion with its antibacterial activities against foodborne pathogens, and characterization of bioactive metabolites: an *in vitro*-*in silico* approach. Front. Microbiol. 11:823. doi: 10.3389/fmicb.2020.00823, 32477292 PMC7237743

[ref3] Arora N. Goldhaber S. Z. (2006). Anticoagulants and transaminase elevation. Circulation 113, e698–e702. doi: 10.1161/circulationaha.105.603100, 16618822

[ref4] Bai Y. Shang Z. Hu R. Gao X. Zhang Z. Li B. . (2025). Study on the antibacterial effect of the new anti-biofilm inhibitor Icac on *Escherichia coli*. Front. Cell. Infect. Microbiol. 15:1710407. doi: 10.3389/fcimb.2025.1710407 PMC12728033 41450570

[ref5] Bai Y. Zhang Z. Xu J. Hu R. Shang Z. Wei X. . (2026). Isoferulic acid suppresses *Escherichia coli* biofilm formation via LuxS/ai-2 quorum sensing inhibition and synergizes with fosfomycin. Front. Microbiol. 17:1837128. doi: 10.3389/fmicb.2026.1837128 PMC13265544 42305676

[ref6] Balewski Ł. Szulta S. Jalińska A. Kornicka A. (2021). A mini-review: recent advances in coumarin-metal complexes with biological properties. Front. Chem. 9:781779. doi: 10.3389/fchem.2021.781779 PMC8671816 34926402

[ref7] Ballén V. Cepas V. Ratia C. Gabasa Y. Soto S. M. (2022). Clinical *Escherichia coli*: from biofilm formation to new antibiofilm strategies. Microorganisms 10:1103. doi: 10.3390/microorganisms10061103, 35744621 PMC9229135

[ref8] Binder D. Probst C. Grünberger A. Hilgers F. Loeschcke A. Jaeger K. E. . (2016). Comparative single-cell analysis of different *E. coli* expression systems during microfluidic cultivation. PLoS One 11:e0160711. doi: 10.1371/journal.pone.0160711, 27525986 PMC4985164

[ref9] Bonnie L. Bassler M. Wright M. R. Silverman (2006). Multiple signalling systems controlling expression of luminescence in *Vibrio harveyi*: sequence and function of genes encoding a second sensory pathway. Molecular Microbiology. doi: 10.1111/j.1365-2958.1994.tb00422.x 7984107

[ref10] Bridges D. F. Rane B. Wu V. C. H. (2018). The effectiveness of closed-circulation gaseous chlorine dioxide or ozone treatment against bacterial pathogens on produce. Food Control 91, 261–267. doi: 10.1016/j.foodcont.2018.04.004

[ref11] Ciofu O. Moser C. Jensen P. Ø. Høiby N. (2022). Tolerance and resistance of microbial biofilms. Nat. Rev. Microbiol. 20, 621–635. doi: 10.1038/s41579-022-00682-4, 35115704

[ref12] Costerton J. W. Stewart P. S. Greenberg E. P. (1999). Bacterial biofilms: a common cause of persistent infections. Science 284, 1318–1322. doi: 10.1126/science.284.5418.1318, 10334980

[ref13] Da Fonsêca D. V. Rocha J. S. Da Silva P. R. De Sá Novaes Pereira H. N. Dos Santos L. V. N. De Santana M. A. D. . (2025). 4-hydroxycoumarin exhibits antinociceptive and anti-inflammatory effects through cytokine modulation: an integrated *in silico* and *in vivo* study. Int. J. Mol. Sci. 26:2788. doi: 10.3390/ijms26062788 PMC11943305 40141429

[ref14] Davin-Regli A. Pagès J.-M. Vergalli J. (2024). The contribution of porins to enterobacterial drug resistance. J. Antimicrob. Chemother. 79, 2460–2470. doi: 10.1093/jac/dkae265, 39205648

[ref15] Fernandez-Ciruelos B. Potmis T. Solomin V. Wells J. M. (2023). Cross-talk between Qsebc and Pmrab two-component systems is crucial for regulation of motility and colistin resistance in enteropathogenic *Escherichia coli*. PLoS Pathog. 19:e1011345. doi: 10.1371/journal.ppat.1011345, 38060591 PMC10729948

[ref16] Flemming H. C. Wingender J. (2010). The biofilm matrix. Nat. Rev. Microbiol. 8, 623–633. doi: 10.1038/nrmicro2415, 20676145

[ref17] Garg S. Gupta J. Sharma S. Sahu D. (2020). An insight into the therapeutic applications of coumarin compounds and their mechanisms of action. Eur. J. Pharm. Sci. 152:105424. doi: 10.1016/j.ejps.2020.105424 32534193

[ref18] Guo J. Xu J. Chen L. Chen Z. Hu H. Nie J. . (2023). Role of Sirt2 in intestinal barrier under cold exposure. Life Sci. 330:121949. doi: 10.1016/j.lfs.2023.121949, 37495079

[ref19] Hawas S. Verderosa A. D. Totsika M. (2022). Combination therapies for biofilm inhibition and eradication: a comparative review of laboratory and preclinical studies. Front. Cell. Infect. Microbiol. 12:850030. doi: 10.3389/fcimb.2022.850030 PMC8915430 35281447

[ref20] He Z. Wang Q. Hu Y. Liang J. Jiang Y. Ma R. . (2012). Use of the quorum sensing inhibitor furanone C-30 to interfere with biofilm formation by Streptococcus mutans and its luxS mutant strain. Int. J. Antimicrob. Agents 40, 30–35. doi: 10.1016/j.ijantimicag.2012.03.016, 22578766

[ref21] Høiby N. Bjarnsholt T. Givskov M. Molin S. Ciofu O. (2010). Antibiotic resistance of bacterial biofilms. Int. J. Antimicrob. Agents 35, 322–332. doi: 10.1016/j.ijantimicag.2009.12.011, 20149602

[ref22] Hufnagel D. A. Depas W. H. Chapman M. R. (2015). The biology of the *Escherichia coli* extracellular matrix. Microbiol. Spectrum 3:1128. doi: 10.1128/microbiolspec.mb-0014-2014, 26185090 PMC4507285

[ref23] Jamal M. Ahmad W. Andleeb S. Jalil F. Imran M. Nawaz M. A. . (2018). Bacterial biofilm and associated infections. J. Chin. Med. Assoc. 81, 7–11. doi: 10.1016/j.jcma.2017.07.012, 29042186

[ref24] Jeon H. Kim Y. G. Lee J. H. Lee J. (2025). Antibiofilm activities of halogenated pyrimidines against enterohemorrhagic *Escherichia coli* O157:H7. Int. J. Mol. Sci. 26:1386. doi: 10.3390/ijms26031386, 39941153 PMC11818689

[ref25] Kalia V. C. Patel S. K. S. Kang Y. C. Lee J.-K. (2019). Quorum sensing inhibitors as antipathogens: biotechnological applications. Biotechnol. Adv. 37, 68–90. doi: 10.1016/j.biotechadv.2018.11.006, 30471318

[ref26] Karygianni L. Ren Z. Koo H. Thurnheer T. (2020). Biofilm matrixome: extracellular components in structured microbial communities. Trends Microbiol. 28, 668–681. doi: 10.1016/j.tim.2020.03.016 32663461

[ref27] Kasperkiewicz K. Ponczek M. B. Owczarek J. Guga P. Budzisz E. (2020). Antagonists of vitamin K—popular coumarin drugs and new synthetic and natural coumarin derivatives. Molecules 25:1465. doi: 10.3390/molecules25061465, 32213944 PMC7146486

[ref28] Kuo W.-T. Zuo L. Odenwald M. A. Madha S. Singh G. Gurniak C. B. . (2021). The tight junction protein zo-1 is dispensable for barrier function but critical for effective mucosal repair. Gastroenterology 161, 1924–1939. doi: 10.1053/j.gastro.2021.08.047, 34478742 PMC8605999

[ref29] Lee J. H. Kim Y. G. Cho H. S. Ryu S. Y. Cho M. H. Lee J. (2014). Coumarins reduce biofilm formation and the virulence of *Escherichia coli* O157:H7. Phytomedicine 21, 1037–1042. doi: 10.1016/j.phymed.2014.04.008, 24837471

[ref30] Limoli D. H. Jones C. J. Wozniak D. J. (2015). Bacterial extracellular polysaccharides in biofilm formation and function. Microbiol. Spectrum 3:1128. doi: 10.1128/microbiolspec.MB-0011-2014, 26185074 PMC4657554

[ref31] Luchini A. C. Rodrigues-Orsi P. Cestari S. H. Seito L. N. Witaicenis A. Pellizzon C. H. . (2008). Intestinal anti-inflammatory activity of coumarin and 4-hydroxycoumarin in the trinitrobenzenesulphonic acid model of rat colitis. Biol. Pharm. Bull. 31, 1343–1350. doi: 10.1248/bpb.31.1343, 18591772

[ref32] Mohapatra S. S. Dwibedy S. K. Padhy I. (2021). Polymyxins, the last-resort antibiotics: mode of action, resistance emergence, and potential solutions. J. Biosci. 46:85. doi: 10.1007/s12038-021-00209-8, 34475315 PMC8387214

[ref33] Nemati A. Dadvar A. Eppinger M. Karimpour Z. Saberi Kakhki S. Sabeti Moghaddam Sabzevar A. . (2025). Shiga toxin-producing *Escherichia coli* (STEC) in developing countries: a 10-year review with global perspective. Microorganisms 13:1529. doi: 10.3390/microorganisms13071529, 40732038 PMC12300086

[ref34] Olaitan A. O. Morand S. Rolain J.-M. (2014). Mechanisms of polymyxin resistance: acquired and intrinsic resistance in bacteria. Front. Microbiol. 5:643. doi: 10.3389/fmicb.2014.00643 PMC4244539 25505462

[ref35] O'toole G. A. (2011). Microtiter Dish Biofilm Formation Assay. Cambridge: MYJoVE Corporation. 10.3791/2437 PMC3182663 21307833

[ref36] Park H. Lee K. Yeo S. Shin H. Holzapfel W. H. (2017). Autoinducer-2 quorum sensing influences viability of *Escherichia coli* O157:H7 under osmotic and *in vitro* gastrointestinal stress conditions. Front. Microbiol. 8:1077. doi: 10.3389/fmicb.2017.01077, 28659895 PMC5468425

[ref37] Rost S. Fregin A. Ivaskevicius V. Conzelmann E. Hörtnagel K. Pelz H.-J. . (2004). Mutations in Vkorc1 cause warfarin resistance and multiple coagulation factor deficiency type 2. Nature 427, 537–541. doi: 10.1038/nature02214, 14765194

[ref38] Sadan S. Shanmugam P. Pillai Z. S. (2026). Advances in 4-hydroxycoumarin chemistry: functionalization, prominent 4-hydroxycoumarin-based therapeutics and their pharmacological significance. Mini Rev. Med. Chem. 26, 911–933. doi: 10.2174/0113895575412635251009051729, 41185509

[ref39] Sambrook J. Russell D. R. (2016). Molecular Cloning: A Laboratory Manual (Third Edition). Cold Spring Harbor: Cold Spring Harbor Laboratory Press.

[ref40] Sauer K. Stoodley P. Goeres D. M. Hall-Stoodley L. Burmølle M. Stewart P. S. . (2022). The biofilm life cycle: expanding the conceptual model of biofilm formation. Nat. Rev. Microbiol. 20, 608–620. doi: 10.1038/s41579-022-00767-0, 35922483 PMC9841534

[ref41] Schauder S. Shokat K. Surette M. Bassler B. (2001). The LuxS family of bacterial autoinducers: biosynthesis of a novel quorum-sensing signal molecule. Mol. Microbiol. 41, 463–476. doi: 10.1046/j.1365-2958.2001.02532.x 11489131

[ref42] Schroeder C. M. Zhao C. Debroy C. Torcolini J. Zhao S. White D. G. . (2002). Antimicrobial resistance of *Escherichia coli* O157 isolated from humans, cattle, swine, and food. Appl. Environ. Microbiol. 68, 576–581. doi: 10.1128/AEM.68.2.576-581.2002, 11823193 PMC126736

[ref43] Schwartz D. J. Chen S. L. Hultgren S. J. Seed P. C. (2011). Population dynamics and niche distribution of Uropathogenic *Escherichia coli* during acute and chronic urinary tract infection. Infect. Immun. 79, 4250–4259. doi: 10.1128/IAI.05339-11, 21807904 PMC3187256

[ref44] Shifflett D. E. Clayburgh D. R. Koutsouris A. Turner J. R. Hecht G. A. (2005). Enteropathogenic *E. coli* disrupts tight junction barrier function and structure *in vivo*. Lab. Investig. 85, 1308–1324. doi: 10.1038/labinvest.3700330, 16127426

[ref45] Solano C. Echeverz M. Lasa I. (2014). Biofilm dispersion and quorum sensing. Curr. Opin. Microbiol. 18, 96–104. doi: 10.1016/j.mib.2014.02.008, 24657330

[ref46] Sun H. Wang M. Liu Y. Wu P. Yao T. Yang W. . (2022). Regulation of flagellar motility and biosynthesis in enterohemorrhagic *Escherichia coli* O157:H7. Gut Microbes 14:2110822. doi: 10.1080/19490976.2022.2110822, 35971812 PMC9387321

[ref47] Suzuki S. Morita Y. Ishige S. Kai K. Kawasaki K. Matsushita K. . (2024). Effects of quorum sensing-interfering agents, including macrolides and furanone C-30, and an efflux pump inhibitor on nitrosative stress sensitivity in *Pseudomonas aeruginosa*. Microbiology 170:001464. doi: 10.1099/mic.0.001464 PMC11263931 38900549

[ref48] Wang Y. Bian Z. Wang Y. (2022). Biofilm formation and inhibition mediated by bacterial quorum sensing. Appl. Microbiol. Biotechnol. 106, 6365–6381. doi: 10.1007/s00253-022-12150-3, 36089638

[ref49] Wang R. Kalchayanand N. King D. A. Brandon E. Bosilevac J. M. Arthur T. M. (2014). Biofilm formation and sanitizer resistance of *Escherichia coli* O157:H7 strains isolated from “high event period” meat contamination. J. Food Prot. 77, 1982–1987. doi: 10.4315/0362-028X.JFP-14-253 25364934

[ref50] Wang Y. Liu B. Grenier D. Yi L. (2019). Regulatory mechanisms of the LuxS/ai-2 system and bacterial resistance. Antimicrob. Agents Chemother. 63:e01186-19. doi: 10.1128/AAC.01186-19, 31383657 PMC6761564

[ref51] Wang H. Wang X. Yu L. Gao F. Jiang Y. Xu X. (2020). Resistance of biofilm formation and formed-biofilm of *Escherichia coli* O157:H7 exposed to acid stress. LWT 118:108787. doi: 10.1016/j.lwt.2019.108787

[ref52] Xu X. Cai M. Lai H. Lian S. Hu L. Cao Y. (2025). Characterization of ai-2/LuxS quorum sensing system in antibiotic resistance, pathogenicity of non carbapenemase-producing carbapenem-resistant *Escherichia coli*. BMC Microbiol. 25:140. doi: 10.1186/s12866-025-03846-3, 40087577 PMC11909908

[ref53] Xu J. Zhang Z. Zhou Y. Zhang H. Shang Z. Dai G. . (2026). Clove oil enhances Fosfomycin efficacy against *Escherichia coli* O157:H7 via biofilm disruption. Biomolecules 16:773. doi: 10.3390/biom16060773, 42352242 PMC13296619

[ref54] Yang Y. Yan X. Redding M. Gu G. Luo Y. Patel J. . (2025). Biofilm formation of *Escherichia coli* O157:H7 strains associated with recent reoccurring lettuce outbreaks. Food Microbiol. 128:104728. doi: 10.1016/j.fm.2025.104728, 39952768

[ref55] Yu Z. Qin W. Lin J. Fang S. Qiu J. (2015). Antibacterial mechanisms of polymyxin and bacterial resistance. Biomed. Res. Int. 2015:679109. doi: 10.1155/2015/679109 PMC4312571 25664322

[ref56] Zhang Z. Xu J. Wei X. Hu R. Zhu Z. Shang Z. . (2025). Antibiofilm inhibitor ferulic acid as an antibacterial synergist against *Escherichia coli*. Biomolecules 15:1253. doi: 10.3390/biom15091253, 41008560 PMC12467221

