# Supplementary material for: 4-Hydroxycoumarin inhibits biofilm formation and enhances antibiotic efficacy against Escherichia coli O157:H7
Source: Front Microbiol. 2026 Oct 1;17:1934000. doi: 10.3389/fmicb.2026.1934000 (PMC13669101; doi:10.3389/fmicb.2026.1934000)
Supplement: Supplementary file 1 [file Supplementary_file_1.DOCX]

**Figure S1.** Growth curves (OD600) of *E. coli* O157 treated with different concentrations of 4-Hydroxycoumarin (100 µg/mL, 200 µg/mL, and 400 µg/mL) or untreated (Control). The interaction between time and group was no significant (*p* = 0.1528). Data are presented as mean ± SEM (n=3).

**Figure S2.** Growth curves (CFU) of *E. coli* O157 treated with different concentrations of 4-Hydroxycoumarin (100µg/mL, 200µg/mL, and 400µg/mL) or untreated (Control). The interaction between time and group (*p* = 0.3878) was no significant. Data are presented as mean ± SEM (n=3).
